# Integrated solutions to manage new normals in healthcare-lessons from corona virus disease 2019

**DOI:** 10.1016/j.amsu.2020.12.037

**Published:** 2021-01-08

**Authors:** Sachet Dawar, Namita Bhutani, D.P.S. Sudan, Sonali Saini, Prem Kumar Singhal, Mohit Bhardwaj, Ayush Pandey, Adil Jokhi Dara

**Affiliations:** a Deptt. of Pulmonary Medicine, SGT Medical College & University, Gurugram, Haryana, India; b Deptt. of Pathology, North DMC Medical College and Hindu Rao Hospital, Delhi, India

**Keywords:** Pandemic preparedness, Technology, COVID-19, Artificial intelligence

## Abstract

Global outbreak of the corona virus disease 2019 (COVID19) has not only challenged the existing healthcare systems but is also a threat to the world economy and stability. In the recent times the world has seen the best healthcare systems collapsing due to the overwhelming burden. Thus, it shows that there have been major lacunae in the pandemic preparedness across the globe. Hence, there is an urgent need to identify the problems, learn from failures and to prepare for future pandemics to reduce the loss of lives and livelihood. In the modern era, blend of public healthcare systems, medical sciences and technology can be put to use to provide solutions. In this article, the authors propose a developmental model of international integrated database software which would connect all the players involved in the management of a pandemic. Better networking and warning system is a key to successful containment of a new viral outbreak.

## Highlights

• COVID-19 has wreaked havoc and even countries with the best healthcare systems have not been able to contain this overwhelming infection.
• Economic losses amounting to trillions of dollars and an uncertain future have triggered an urgent requirement of novel solutions for such novel infections.
• As per our understanding, the failure to contain this public health emergency lies in the poor pandemic preparedness beforehand and a lack of dedicated integrated national and international system.
• A new artificial intelligence technology backed management system is required to deal with such scenarios in future to enable efficient risk stratification and optimized resource allocation.
• Technology companies should take initiatives to build such software to solve problems being faced now for a better future.

## 1 Background

In December 2019, a cluster of acute respiratory illness, now known as Corona virus disease 2019 (COVID 19), occurred in Wuhan, Hubei Province, China [1]. Full-genome sequencing and phylogenetic analysis indicated that novel corona virus 2019 is a distinct clad from the beta-corona viruses which are associated with severe acute respiratory syndrome (SARS) and Middle East respiratory syndrome (MERS) [2].

Till December 2020 more than 76.8 million individuals were affected and more than 1.69 million people have lost their lives to COVID-19 [3]. Globally, the disease has wreaked havoc and even countries with the best healthcare systems have not been able to contain this overwhelming infection. Economic losses amounting to trillions of dollars and an uncertain future have triggered an urgent requirement of novel solutions for such novel infections [4].

National wide measures were implemented including local lockdown to control viral spread but as per our understanding, the failure to contain this public health emergency lies in the poor pandemic preparedness beforehand and a lack of dedicated integrated national and international system. A new artificial intelligence technology backed management system is required to deal with such scenarios in future to enable efficient risk stratification and optimized resource allocation.

## 2 International integrated management system (IIMS) for COVID 19 like pandemics

The proposed integrated management system would be an international online database of viral diseases and their management. It would be an ultimate amalgamation of virology, zoology, laboratory & clinical medical sciences, information technology, drugs & equipments manufacturing industry and government resources (epidemiological and geographical data).

The database can be compiled and selectively accessed in the form of a mobile application for easy accessibility. Formation of such database would require immense investment on all fronts, from research for all possible pathogenic viruses – their identification, structure, mode of spread, pathogenesis of disease, prevention, vaccines, diagnostic facilities, possible treatments to equipments required in management and logistical support. Artificial intelligence and data sciences would definitely be required to aid human intelligence in this mammoth networking project and its regular update as new researches keep coming on the worldwide web.

**Fig. 1:**
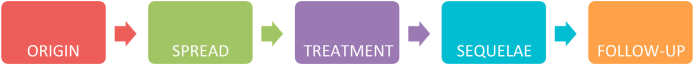
Flow chart of sequence of events in a viral disease and its management.

To find out possible check points for intervention we must understand the sequence of events linked to a viral disease and its management (Fig. 1).

## 3 Check points for intervention via IIMS

### 3.1 Origin

World over, scientists at virology institutes maintain records and specimen of previously reported viral species and their genomic data for research purposes. Such data should be made available on the IIMS for the global virology community. For any new zoonotic or human viral infection, the data regarding its discovery and genomic codes must be made available on this platform. Software must immediately sound an alarm with the government authorities on the discovery of novel viral species or strain.

Virologists, zoologists, microbiologists and veterinarians must work in close association to identify the new viruses or the new mutations in the already occurring strains. So, an integrated platform would be of immense help to share, learn and apply the knowledge.

### 3.2 Spread

Containment of the infection in a particular geographical area is the biggest challenge for any government especially for viruses, which are known to be highly contagious. This involves trained man power to identify and report the geographical spread. N 95 and surgical masks have been used all over the world to control the spread of infections. Now, mobile applications like Aarogya setu [5] have set new benchmarks in identifying suspects and notifying the government authorities. Such applications if linked to the IIMS database would prove to be highly beneficial.

For diagnosis of any new virus affecting humans or animal, newer diagnostic kits have to be developed. If diagnostic kits manufacturers have an early access to the genome data through the IIMS they can speed up the process and save lot of time. Also, they can receive feedback from the end user and make necessary modifications and work upon quality assurance.

Active surveillance for early identification of cases and early isolation can help the government authorities to rapidly establish containment zones and cordon off the area to public access. Current gold standard for the etiological diagnosis of SARS-CoV-2 infection is real time reverse transcription polymerase chain reaction (rRT-PCR) on respiratory tract specimens [6]. A major fallacy in the COVID-19 pandemic that was faced by many countries was the acute shortage of diagnostic kits.

Vaccines are an important tool in containing case fatalities [7]. Vaccine development for any new virus is a challenge. IIMS database would allow researchers to share and access information about the vaccine trials for global community. Bulk manufacturers must keep the government authorities updated with the quantity of stocks, distribution and manufacturing capacities.

Hence, national and international authorities through the IIMS can have up to date information of the available diagnostic kits, vaccines, their stocks, validity and distribution across the countries.

### 3.3 Treatment

Treating contagious viral illnesses requires separate isolation centres, intensive care units, equipments, trained medical and paramedical staffs with fully equipped labs and all diagnostic modalities and respiratory and therapeutic devices. Dedicated infectious diseases hospitals should be established at district level to handle such public health emergencies. IIMS would keep updated records of the available staffs, bed strength and other valuable resources for timely access and allocation by the government authorities. These hospitals will prevent the community transmission through healthcare systems, thereby interrupting the course of epidemic. Healthcare system preparedness is a major aspect to be worked upon and has been a major failure in the COVID-19 pandemic even in the countries with best healthcare system in the world.

Any new virus demands rapid research and development of newer anti-viral drugs and trials of existing drugs and therapeutic modalities to know the efficacy against the novel pathogen. Since the onset of COVID-19, researchers have launched more than 180 clinical trials varying from repurposed antivirals and immunomodulators to unproven plasma cell therapies and many further trials are in the pipeline [8]. This process involves huge investments in clinical trials and generates lots of data which is not available on a single platform making it difficult to access. By using the IIMS and the artificial intelligence technology, the drug trial data would be analysed faster and can be used at a global platform. Also, the manufacturers must update the drug stocks, manufacturing capacity and geographical outreach on the database for efficient allocation by the government authorities to meet the demand supply deficit.

### 3.4 Sequelae

The management of viral illnesses is not limited to the treatment of the disease but it is an ongoing process to manage the sequelae as well. Depending upon the organs involved, the sequalae occur. From minor immune disturbances to widespread pulmonary fibrosis, sequelae can have a detrimental impact on quality of life. Dedicated mass scale rehabilitation centres should be setup which focuses on functional as well as mental well being of the patient. The IIMS.

Database would have the locations and availability of equipments at such rehabilitation centres and would also serve as a platform to connect to the concerned staffs.

### 3.5 Follow up

Regular follow-up of recovered patients must be done to assess outcomes of any management protocol. It generates a lot of data pertaining to their mental and physical status, quality of life, experience with the healthcare facilities etc. This data is valuable for research purposes and improvement of the healthcare system. A platform like IIMS will be able to accumulate research data by the help of artificial intelligence for analysis and thus give a boost to pandemic management in future.

## 4 Discussion

The World Health Organization (WHO) and its member countries act according to their respective pandemic management guidelines which are well researched and regularly updated. Despite such guidelines and management protocols, the situation in COVID-19 has posed difficult challenges and has shown that even the best healthcare systems can collapse. Delayed warning, lack of co-ordination, poor allocation of resources and non-specific treatment are some of the factors responsible.

**Fig. 2:**
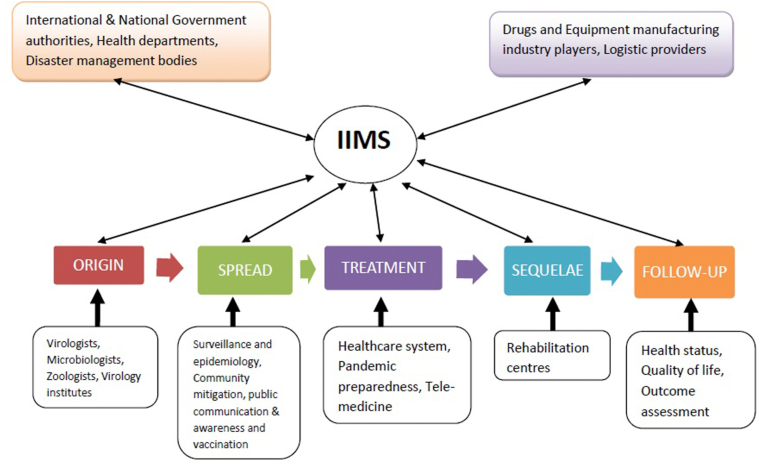
Diagrammatic representation of networking through IIMS platform at various events in a pandemic.

An international integrated management system can be a solution to many of the problems being faced in the current crisis. It can bring together all the major players on a single platform. Scientists, academicians, lab experts and industry experts would be welcomed for regularly updating the existing database. The database would be available for all identified viral pathogens and guidelines can be made available keeping in mind geographical and climate variation across the globe. Co-ordination with industry is essential to maintain a smooth and continuous supply of all essential medical equipments like masks, personal protective equipments, N 95 masks, Surgical and other masks, gloves and other essential medical supplies globally (Fig. 2).

Incorporation of artificial intelligence (AI) to medical sciences would definitely help in dealing with huge amounts of data and also reduce man power requirements. In 2016, the biggest amount of investments in AI research was in healthcare applications as compared with other sectors [9]. AI in medicine can be dichotomized into two subtypes: virtual and physical. The virtual part ranges from applications such as electronic health record systems to neural network-based guidance in treatment decisions which could lay down the foundation of our proposed software project. The physical AI like robots assisting doctors or patients gives added advantage to tele-medicine, which can help in remote areas [10].

## 5 Limitations

As per our understanding, following limitations could deter the development of IIMS software:

1. Huge financial and infrastructure investment.
2. Inadequate government support.
3. Intellectual property rights (IPR) issues.
4. International disagreements.

## 6 Conclusion

Hence, we conclude that tough lessons should be learnt from COVID-19 pandemic and the world has to be prepared with novel solutions for future pandemics to minimize the economic and human losses. Technology companies should take initiatives to build such software to solve problems being faced now for a better future.

## Source(s) of support in the form of grants, equipment, drugs, or all of these

None.

## Ethical approval

Not applicable.

## Sources of funding

Nil.

## Author contribution

1. Dr. Sachet Dawar and Dr. Namita Bhutani: STUDY CONCEPT AND WRITING THE MANUSCRIPT
2. Dr. DPS Sudan and Dr. Sonali Saini: DATA COLLECTION
3. Dr. Prem Kumar Singhal and Dr. Mohit Bhardwaj: DATA ANALYSIS
4. Dr. Ayush Pandey and Dr. Adil Jokhi Dara: Software work

## Trail registry number

Not applicable.

## Guarantor

DR. Sachet Dawar.

## Registration of Research Studies

Not applicable.

## Consent

Not applicable.

## Declaration of competing interest

Nil.
